# LRP5 promotes cancer stem cell traits and chemoresistance in colorectal cancer

**DOI:** 10.1111/jcmm.17164

**Published:** 2022-01-07

**Authors:** Xiaobo Nie, Huiyang Liu, Wenling Ye, Xiaoyun Wei, Lili Fan, Han Ma, Lanqing Li, Wanting Xue, Wenting Qi, Yan‐Dong Wang, Wei‐Dong Chen

**Affiliations:** ^1^ Key Laboratory of Receptors‐Mediated Gene Regulation and Drug Discovery School of Basic Medical Sciences People’s Hospital of Hebi Henan University Henan China; ^2^ State Key Laboratory of Chemical Resource Engineering College of Life Science and Technology Beijing University of Chemical Technology Beijing China; ^3^ Key Laboratory of Molecular Pathology School of Basic Medical Science Inner Mongolia Medical University Inner Mongolia China

**Keywords:** cancer stem cells, canonical Wnt/β‐catenin signalling pathway, colorectal cancer, IL‐6/STAT3 signalling pathway, LRP5

## Abstract

The overactivation of canonical Wnt/β‐catenin pathway and the maintenance of cancer stem cells (CSCs) are essential for the onset and malignant progression of most human cancers. However, their regulatory mechanism in colorectal cancer (CRC) has not yet been well demonstrated. Low‐density lipoprotein receptor‐related protein 5 (LRP5) has been identified as an indispensable co‐receptor with frizzled family members for the canonical Wnt/β‐catenin signal transduction. Herein, we show that activation of *LRP5* gene promotes CSCs‐like phenotypes, including tumorigenicity and drug resistance in CRC cells, through activating the canonical Wnt/β‐catenin and IL‐6/STAT3 signalling pathways. Clinically, the expression of LRP5 is upregulated in human CRC tissues and closely associated with clinical stages of patients with CRC. Further analysis showed silencing of endogenous *LRP5* gene is sufficient to suppress the CSCs‐like phenotypes of CRC through inhibiting these two pathways. In conclusion, our findings not only reveal a regulatory cross‐talk between canonical Wnt/β‐catenin signalling pathway, IL‐6/STAT3 signalling pathway and CD133‐related stemness that promote the malignant behaviour of CRC, but also provide a valuable target for the diagnosis and treatment of CRC.

## 1 INTRODUCTION

Colorectal cancer (CRC) is the third most common cancer and the fourth leading cause of cancer‐related mortality worldwide, with almost 1.93 million newly diagnosed cases and 935,000 deaths occurred worldwide in 2020.^1^ The routine use of colonoscopy and systematic therapies, such as chemo‐ and radiation therapy as well as immunotherapy have effectively improved the diagnosis rate, controlled many localized CRC and evidently improved clinical prognosis.^2^ However, when it progresses to advanced stages, because of the metastasis, acquisition of chemoresistance and recurrence, the 5‐year survival rate sharply declines to be less than 30%. Therefore, it is imperative to explore accurate biomarkers and elucidate mechanisms underlying tumorigenesis of CRC for developing novel and more effective therapeutic strategies.

As a small subpopulation of cancer‐initiating cells, cancer stem cells (CSCs) will undergo asymmetric division to maintain their own pool and differentiate into ordinary common tumour cells and are considered being responsible for the initiation, maintenance, progression, metastasis, relapse, chemo‐/radioresistance and other phenotypes of many types of human malignancies, including CRC.^3^, ^4^, ^5^ CSCs in CRC were first reported in 2007 that CRC was created and propagated by a rare population of undifferentiated tumorigenic cells expressing CD133.^6^, ^7^ Subsequently, a series of studies showed that the maintenance of colorectal CSCs‐like phenotypes was also closely related to the specifical expression of other protein markers such as aldehyde dehydrogenase (ALDH1A1), Lgr5, Nanog and Bmi1, and the overactivation of several important signalling pathways including Notch, OCT3/4, Hedgehog and Wnt *etc*.^8^, ^9^, ^10^, ^11^ Among them, the hyperactivation of Wnt pathways is considered being one of the most important events for the occurrence and progression of a variety of cancers. Specifically, activation of canonical Wnt/β‐catenin via upregulation of its key components β‐catenin and c‐Myc could augment the formation of CRC cellular colonospheres, which are particularly concentrated in CSCs.^12^ Consistent with these observations, hypoxia‐induced canonical Wnt/β‐catenin/Id2 cascade increased the occurrence of CSCs‐like phenotypes of CRC cells.^13^ Conversely, blocking the canonical Wnt pathway by phenethyl isothiocyanate, histone demethylase inhibitor JIB‐04 or its inhibitor IC‐2 could effectively inhibit colorectal CSCs and sensitize CRC cells to chemotherapeutic agents.^14^, ^15^, ^16^ However, the underlying mechanisms of canonical Wnt/β‐catenin pathway dysregulation in colorectal CSCs have not been clearly elucidated.

Low‐density lipoprotein receptor‐related protein 5 (LRP5) serves as a key co‐receptor with Frizzled (FZD) proteins for transmitting signals by Wnt ligands.^17^ The canonical Wnt/β‐catenin pathway is activated upon binding of secreted Wnt ligands to the FZD and LRP5/6; then, Dishevelled (DVL) proteins are activated and recruited to form DVL polymers that can deactivate the ‘destruction complex’ composed of Axin, adenomatous polyposis coli (APC) and glycogen synthase kinase 3β (GSK3β), thereby β‐catenin is stabilized and translocated into nucleus to form β‐catenin‐LEF/TCF transcriptional complex complexes, thus to activate downstream oncogenes such as *CCND1* and *c*‐*Myc*, ultimately brings about the development of human malignancies.^18^, ^19^ Inversely, in the absence of Wnt ligands, β‐catenin is sequestered by the ‘destruction complex’ and prone to be phosphorylate followed by the degradation via ubiquitination, which ultimately inactivates the β‐catenin mediated Wnt signalling. LRP5 has been proved to be less effective than LRP6 in transducing Wnt signal, perhaps owing to the fact that the PPPSPxS motifs, which are the cytoplasmic domains of LRP5/6 and provide inducible docking sites for Axin, are easier to be phosphorylated in LRP6 than in LRP5.^20^, ^21^ However, mutation or polymorphism of *LRP5* gene is still closely associated with the defect of tissue homeostasis and pathogenesis of multiple diseases.^22^, ^23^, ^24^, ^25^ The aberrant expression of LRP5 has been reported to participate in the development of several human malignancies.^26^, ^27^, ^28^, ^29^ However, its role in the progression of different cancers has shown some discrepancies and needs to be determined in specific type of cancers. At present, the expression pattern and role of LRP5 in CRC have not been documented and urgently need investigation.

Here, we report LRP5 plays a vital oncogenic role in CRC development and contributes to the chemoresistance of CRC cells, partially through activating the canonical Wnt/β‐catenin and IL‐6/STAT3 signalling pathways and consequently promoting CSCs phenotypes. Clinically, LRP5 is overexpressed in CRC tissues and some corresponding cell lines, the expression of endogenous *LRP5* gene is closely associated with that of CSCs‐related gene *PROM1* (encoding CD133) and represents a valuable diagnostic biomarker for CRC. Moreover, silencing the endogenous *LRP5* gene significantly inhibits the tumorigenicity of CRC, increases the chemosensitivity and apoptosis of CRC cells. Therefore, our study proposed a novel insight into the mechanism of CRC and provides a promising biomarker and target for the diagnosis and treatment of CRC.

## 2 MATERIAL AND METHODS

### 2.1 Immunohistochemistry (IHC)

The CRC tissue microarray embedded in paraffin was purchased from Shanghai Outdo biotech (Shanghai, China), and IHC was carried out as described. In brief, the section was deparaffinized by incubation at 65°C for 1 h and further rehydrated through graded ethanols, and the antigen retrieval was performed by boiling the section for 30 min in 1‐mM EDTA buffer (pH 7.5), followed by treated with 3% H_2_O_2_ to quench the endogenous peroxidase. After incubation with 5% sheep serum albumin for 30 min to block the nonspecific binding, the section was incubated overnight with a primary antibody against LRP5 protein (Abcam, UK) (1:500) at 4°C; after rinsed with PBS for three times, the section was then incubated with the secondary antibody for 30 min at 37°C. Finally, the section was incubated with 3′3‐diaminobenzidine tetra‐hydrochloride (DAB) to develop colour and then counterstained with haematoxylin. Meanwhile, IHC images of LRP5 protein expression in normal colorectal tissues and CRC ones were downloaded and examined from the Human Protein Atlas (HPA) (http://www.proteinatlas.org/).

### 2.2 Collection of colorectal tissue specimens

All used human colorectal samples were obtained from the Department for General Surgery, Affiliated Huaihe Hospital of Henan University on CRC patients who underwent enterectomy in 2017. The patients were selected based on clinical‐pathological parameters of CRC and none of them received preoperative anticancer treatments. All tissues were snap‐frozen immediately and always kept in liquid nitrogen until further analysis. The collection of colorectal tissue samples and related clinical data was approved by the Biomedical Research Ethics Committee of Henan University, Kaifeng.

### 2.3 Cell culture and fluorescence‐activated cell sorting (FACS)

Human CRC cell line HCT‐116 was obtained from the School of Basic Medicine, Peking Union Medical College (Beijing, China) and maintained in Dulbecco's Modified Eagle's Medium (Thermo, USA) with 10% foetal bovine serum (FBS) (Thermo, USA) and 1% penicillin/streptomycin (Gibco, USA) in a humidified atmosphere of 5% CO_2_ at 37°C. For cell sorting experiments, HCT‐116 cells were stained with CD133 antibody (Miltenyi Biotec, Germany) at 4°C for 15 min away from light and washed with PBS; then, cells were resuspended with PBS followed by filtrated through sterile 200 mesh cell strainers to separate single cell before sorting. The populations of CD133^+^ and CD133^−^ cells were determined and sorted by MoFlo XDP over‐speed flow cytometry sorter (Beckman, USA), selected cells were propagated, and the expression of CD133^+^ was determined by quantitative real‐time PCR (qRT‐PCR).

### 2.4 Selecting stable CRC cells with the activation or knockdown of LRP5

The CRISPR/Cas9 KO plasmid or CRISPR activation plasmid that disrupts or activates the endogenous *LRP5* gene was purchased (Santa Cruz, USA). HCT‐116 cells were plated at a density of 3 × 10^5^ cells per well of a 6‐well plate 24 h before transfection. LRP5‐CRISPR/Cas9 KO plasmid (LRP5‐KO), LRP5‐CRISPR activation plasmid (LRP5‐ACT) or corresponding controls were transfected into cells separately by using lipofectamine 3000 (Invitrogen, USA) in serum‐free medium; then, cells were cultured in medium containing 5 μg/ml puromycin for two weeks, and single cell was selectively by using flow cytometer followed by transferred to 96‐well plate for expansion. Cell clones exhibiting the most efficient downregulation or upregulation of LRP5 compared with corresponding controls were verified by qRT‐PCR and immunoblot analysis and selected for subsequent analysis.

### 2.5 RNA isolation and qRT‐PCR analysis

RNA extraction and qRT‐PCR were performed according to our previous work.^30^ Sequences of primers used for real‐time PCR are listed in Table S1.

### 2.6 Protein extraction and immunoblot

Protein extraction from cells or tumour tissues and immunoblot were carried out according to our previous work.^31^ Specific primary antibodies used were against LRP5 (1:1000), STAT3 (1:1000), p‐STAT3 (1:1000) and β‐actin (1:1000). All primary antibodies were purchased from Cell Signaling Technology (USA). The blots were imaged using SuperSignal West Pico Chemiluminescent Substrates (Thermo, USA), and signals were collected under Automatic Multifunction Chemiluminescent Detection System (Tanon, China); then, the intensity of bands was quantified by using Image‐J Software.

### 2.7 Cell proliferation assays

To evaluate cell proliferation ability, stable cell clones were plated at a density of 4 × 10^3^ cells per well (ACT groups) or 9 × 10^3^ cells per well (KO groups) of E‐plate 16 in a humidified atmosphere of 5% CO_2_ at 37°C. The proliferative indices of cells were recorded every 15 min until 60 h by using the real‐time cellular analysis (RTCA, ACEA Biosciences, USA) system, and cellular proliferative curves of each group were generated based on the mean values of quadruplicate wells. To determine the effect of LRP5 knockdown or activation on the sensitivity of CRC cells to cisplatin treatment, stable LRP5‐KO or LRP5‐ACT HCT‐116 cells and corresponding controls were seeded into the E‐plate 16 and incubated for 24 h, followed by treated with cisplatin at a concentration of 8 μg/ml for 36 h or 12 μg/ml for 24 h. The proliferation index was also recorded by using RTCA system. The inhibition rate of cisplatin on cells was calculated by the equation: [proliferation index (PBS)‐proliferation index (cisplatin)]/proliferation index (PBS) ×100%.

### 2.8 Cell apoptosis analysis

Cellular apoptosis was detected by using Annexin‐V‐FITC/PI apoptosis kit (Multi Sciences, China) according to the instructions. In brief, stable LRP5‐KO or control HCT‐116 cells were cultured into the 60‐mm plates for 24 h and then treated with cisplatin (8 μg/ml) for another 36 h. Cells were then suspended with 500‐μL binding buffer after harvest, followed by added with 5‐μl Annexin‐V‐FITC and 10‐μl PI and incubated for 5 min at room temperature. Apoptosis of cells was analysed by NovoCyte^TM^ flow cytometer (ACEA Biosciences Inc., USA).

To analyse the influence on apoptosis‐related genes, stable LRP5‐KO or LRP5‐ACT HCT‐116 cells and their controls were seeding into 6‐well plate and incubated for 24 h, followed by treated with 8‐μg/ml or 12‐μg/ml cisplatin for another 36 h. Cells were harvested for analysing the mRNA levels of pro‐apoptotic genes, such as *BAX*, *CYCS* (cytochrome C), *CASP3* (caspase 3), *TP53* (p53), *CDKN1A* (p21), *CDKN1B* (p27) and *FAS*.

### 2.9 Wound healing migration assay

Cells were seeded into 6‐well plates (2 × 10^5^ cells/well) and cultured till reaching 80% confluence; then, cells were scraped by a 100‐μl pipette tip. The plate was washed with PBS to remove the floating cells completely and replaced with fresh medium contains 2% FBS. Cells were cultured and photographed every 24 h to monitor the migrated distance. The relative migration rate was calculated by deducting w 48h/96 h (the final width) from w 0 (the initial width) and normalizing to the initial width.

### 2.10 Colony formation assay

A total of 500 cells (ACT groups) or 800 cells (KO groups) per well were seeded into a 6‐well plate and cultured at 37°C for 14 days (ACT groups) or 10 days (KO groups). After washing with PBS for three times and fixed with 75% ethanol, cells were stained with 0.1% crystal violet. The number of colonies was counted automatically by using Image Pro Plus (IPP) software (Media Cybernetics).

### 2.11 Tumoursphere formation

Cells (300 cells/well) were seeded into a 96‐well plate and maintained in serum‐free stem medium consisting of DMEM, B27 (Invitrogen), 20‐ng/ml human epidermal growth factor and 20‐ng/ml human fibroblast growth factor (Thermo, USA), 10‐ng/ml heparin (Sigma, USA), 1% methylcellulose (Sigma, USA) and 1% antibiotics. Tumourspheres that arose within 12 days (ACT groups) or 16 days (KO groups) were recorded under the microscope at 100× magnification and counted by the IPP software. The mean tumoursphere formation efficiency (TSFE) of cells was calculated by the equation: [(number of spheres formed/number of cells seeded) ×100%].

### 2.12 Xenograft tumour assay in nude mice

Female BALB/c nude mice at 4 weeks old were purchased from Beijing Vital River Laboratories (Beijing, China) and fed with standard rodent chow under specific pathogen‐free (SPF) conditions. Mice were subcutaneously injected with 2 × 10^6^ (ACT groups) or 5 × 10^6^ (KO groups) HCT‐116 cells resuspended in 150 μl of serum‐free PBS. Tumour size was measured every three days to estimate tumour volume (length × width × width) using a Vernier calliper. Mice were anaesthetized 24 days later and sacrificed to harvest all tumours. To evaluate the effect of LRP5 activation on the drug resistance of CRC cells to chemotherapeutic agents *in vivo*, the nude mice were implanted with 4 × 10^6^ LRP5‐ACT HCT‐116 cells or control cells resuspended in 150 μl of serum‐free PBS, followed by received i.p. treatment of 1 mg/kg cisplatin every three days from the third day of transplantation. The body weight of the nude mice was measured every three days, and all mice were humanely sacrificed on day 24 and the tumour weight was measured. All animal studies were conducted with approval from the Biomedical Research Ethics Committee of Henan University.

### 2.13 GEO data mining analysis

A retrospective study on the Cancer Genome Atlas (TCGA) Colon and Rectal Cancer (15 data sets) was performed based on the data obtained from the University of California, Santa Cruz (UCSC) Xena browser (https://xenabrowser.net/datapages/) (last access: October 2021). Primary tumour tissues from 380 CRC patients and 51 cases of adjacent normal tissues were collected for gene expression RNA‐seq. Three hundred and sixty‐seven of the 380 CRC patients have overall survival (OS) data, and 380 patients have recurrence‐free survival (RFS) data. Gene expression RNA‐seq for LRP5, PROM1 (CD133) and STAT3 mRNA expression analyses and clinicopathological parameters of CRC patients including gender, pathological grade, TNM stage, body mass index (BMI), history of colon polyps, age at initial pathological diagnosis, vital status, OS and RFS were downloaded for subsequent analysis. Patients with CRC were divided into two groups according to the cut‐off mean value of LRP5 expression. Kaplan‐Meier curves of RFS and OS were drawn to evaluate the difference in survival outcomes between patients with low or high LRP5 expression. The odds ratio (OR) with 95% confidence interval (CI) and *p*‐value for the LogRank test were calculated to evaluate the association between the clinicopathological parameters and RFS/OS in CRC patients.

### 2.14 Statistical analysis

Statistical significance was analysed by SPSS 25.0 software and, figures were drawn using GraphPad Prism 8.0. Two‐sided Student's *t* test was conducted to evaluate the statistical significance of the difference between two groups. Chi‐square tests were performed to assess the correlation between the expression levels of LRP5 and clinicopathological parameters. The mean value of LRP5 expression was set as the cut‐off to draw the Kaplan‐Meier curves of RFS and OS, and the LogRank test was used for evaluating the difference between the survival rates of CRC patients. The univariate survival analysis (Kaplan‐Meier estimates) was carried out to evaluate the prognostic role of LRP5 regarding to RFS and OS for CRC patients by SPSS software, and risk factors with *p* < 0.1 in univariate analysis were further evaluated by multivariate Cox regression analysis. The correlation among the expression of LRP5, PROM1 (CD133) and STAT3 was evaluated by Pearson correlation analysis, and error bar indicates the standard deviation of mean value. A *p*‐value less than 0.05 was considered statistically significant. One, two and three asterisks represent *p*‐values of < 0.05, 0.01 and 0.001 respectively.

## 3 RESULTS

### 3.1 LRP5 is overexpressed in CRC cell lines and tissues

**FIGURE 1:**
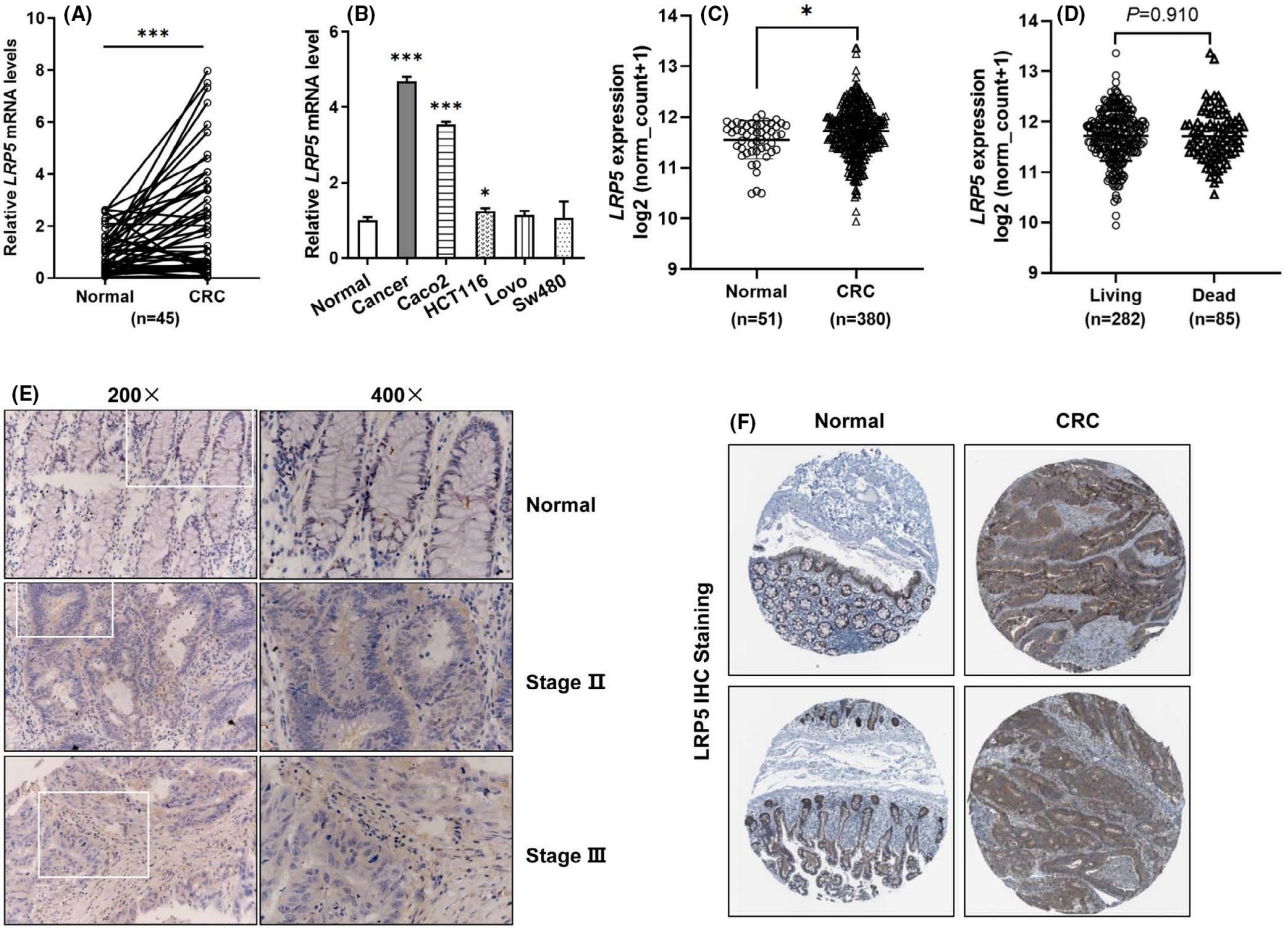
LRP5 expression is upregulated in human CRC tissues and cell lines. (A) The mRNA levels LRP5 in CRC tissues and paired normal tissues were analysed by qRT‐PCR (*n* = 45). (B) LRP5 mRNA levels in different CRC cell lines were examined by qRT‐PCR, compared with normal colorectal tissues. (C) Plot charts of LRP5 mRNA levels in CRC tissues and normal colorectal ones by using the TCGA RNA‐seq data. (D) Plot charts of LRP5 mRNA levels in CRC patients with different living status. (E) Representative LRP5 IHC staining images in normal section and CRC tissue sections in stages II and III (200× and 400×). (F) Representative LRP5 IHC staining images, downloaded from the online human Protein Atlas (HPA) (http://www.proteinatlas.org/), in normal tissues (left) and in CRC tissues (right). *p < 0.05, ***p < 0.001 vs. control group

Several studies have reported that LRP5 is crucial for the tumorigenesis of several types of human malignancies. To determine the expression pattern of LRP5 in CRC microenvironment, the mRNA levels of LRP5 in 45 pairs of CRC tissues and corresponding non‐carcinomatous normal tissues and four commonly used CRC cell lines were analysed by qRT‐PCR. The results showed the mRNA levels of LRP5 were obviously upregulated in CRC tissues (*p *= 0.0009) and Caco2 cells (*p *= 0.0009), and was slightly elevated in HCT116 cells (*p *= 0.047) compared with normal tissues (Figure 1A, B). Importantly, we reconfirmed the results through analysing data from TCGA (Figure 1C). Other oncomine analysis of CRC tissues versus normal ones also proved LRP5 was overexpressed in CRC (Table 1). However, there was no obvious difference in LRP5 mRNA levels in patients with different survival outcomes (Figure 1D). In addition, we performed IHC in tissue microarray containing paired CRC specimens and normal ones. As shown in Figure 1E, a similar upregulation of LRP5 protein was detected in CRC tissues with different pathological grades compared with that in normal colorectal tissues (Figure 1E). Similarly, after analysing the images for IHC staining from the online HPA database, we found CRC tissues had strong LRP5 staining but normal colorectal tissues had low LRP5 staining (Figure 1F).

**TABLE 1:** Data sets of *LRP5* gene expression in CRC from the UCSC Xena browser

Gene	Normal (Cases)	Tumour (Cases)	Fold Change	*t*‐Test	*p*‐Value
LRP5	Colon (32)	Colon adenocarcinoma (222)	1.008	1.105	0.2702
	Colon (41)	Colon adenocarcinoma (469)	1.045	1.968	0.050
	Rectum (10)	Rectum adenocarcinoma (166)	1.16	3.589	0.0004
	Rectum (10)	Rectum adenocarcinoma (94)	1.037	2.721	0.0076

### 3.2 Correlation between the LRP5 mRNA levels and clinicopathological parameters in CRC tissues

**FIGURE 2:**
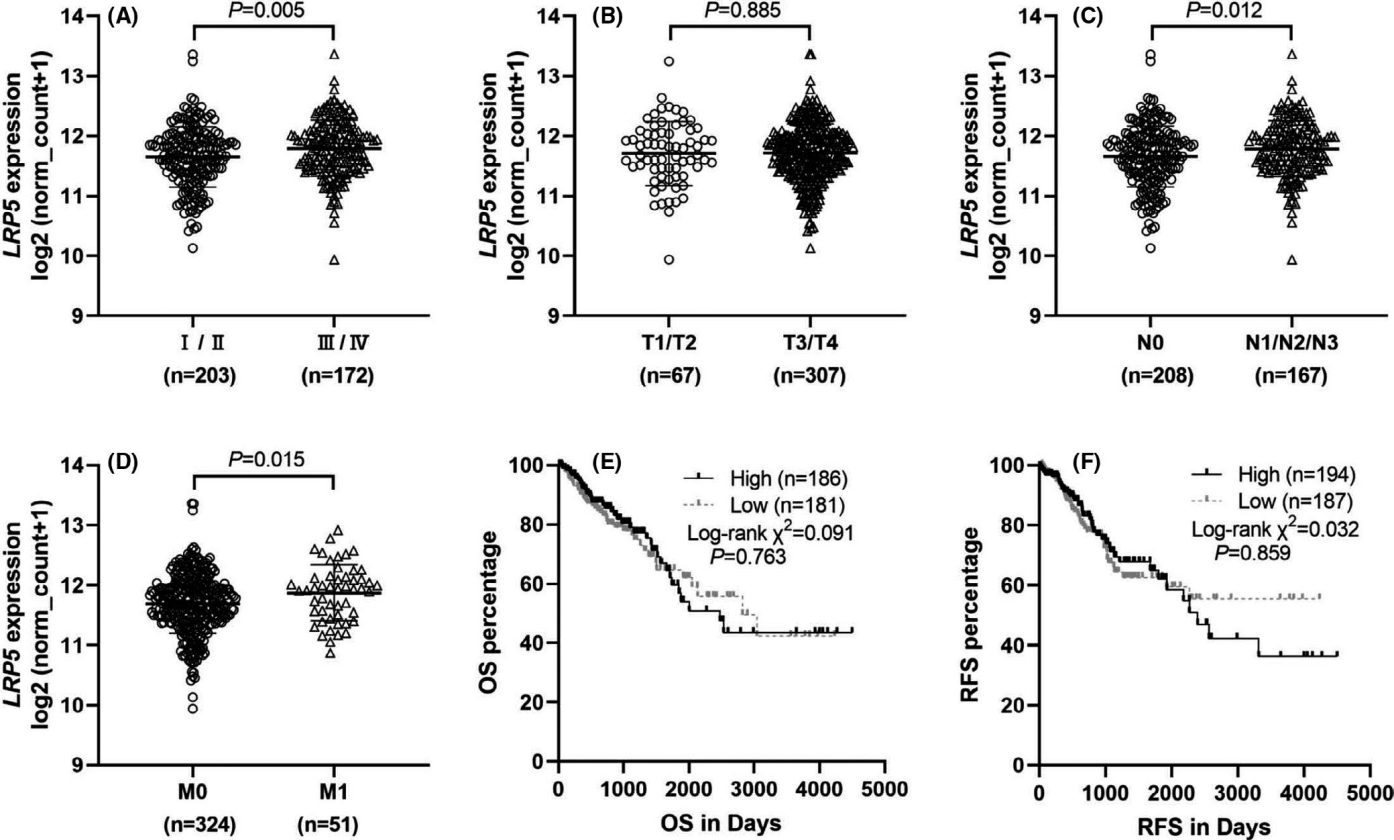
Analysis of LRP5 mRNA levels and various clinicopathological parameters in CRC tissues based on TCGA data. (A‐D) Comparison of LRP5 mRNA levels in CRC patients, according to Ⅰ‐Ⅳ stages (A) and TNM stages (B‐D). (E‐F) Kaplan‐Meier curves of OS (E) and RFS (F) in CRC patients. A *P*‐value lower than 0.05 was considered as statistically significant

The mRNA levels of LRP5 in different pathological stages were further assessed by analysing the data for CRC from TCGA database. The results showed that the LRP5 mRNA level was obviously elevated in advanced stages (stage Ⅲ and stage Ⅳ) compared with that in early stages (stage Ⅰ and stage Ⅱ) (Figure 2A). However, as shown in Figure 2B, no difference in LRP5 mRNA level was found between tumour grade in small (T1 and T2) and tumour grade in large (T3 and T4). CRC tissues with involvement of local lymph node (N1/N2/N3) or metastasis displayed a higher level of LRP5 mRNA level compared with that without involvement of local lymph node or metastasis (Figure 2C, D). To validate the association between LRP5 mRNA level and OS or RFS, we mapped the Kaplan‐Meier curves of OS and RFS and no obvious association was found between them, even though it seemed CRC patients with low LRP5 mRNA level had superior OS and RFS compared with those with high LRP5 mRNA level (Figure 2E, F).

We subsequently assessed the independent prognostic value of LRP5 mRNA level in CRC. The association between the LRP5 expression levels and various clinicopathological characteristics of CRC patients was summarized in Table 2. Interestingly, the high LRP5 expression group showed an obviously higher ratio in patients with distant metastasis (33/157 vs. 18/167; *p* = 0.031) and in advanced stages (stage Ⅲ and stage Ⅳ) (97/93 vs. 75/110; *p* = 0.041) in comparison with the low LRP5 expression group. Furthermore, univariate survival analysis showed that tumour grade in large, involvement of local lymph node, distant metastasis and advanced stages were correlated with inferior OS and RFS for CRC, patients over 65 years of age had inferior OS, and male patients had inferior RFS (Table 3). The subsequent multivariate analysis showed that distant metastasis (OR: 2.403; 95% CI: 1.359–4.248, *p*=0.003; Table 3) and age over 65 years (OR: 2.970; 95% CI: 1.827–4.829, *p *< 0.001; Table 3) were independent prognosis factors in terms of OS, whereas distant metastasis (OR: 2.226; 95% CI: 1.223–4.021, *p *= 0.008; Table 3) and males (OR: 1.534; 95% CI: 0.970–2.426, *p *= 0.05; Table 3) displayed shorter RFS. In short, the above data show LRP5 is overexpressed in CRC tissues and has potential value in diagnosing CRC.

**TABLE 2:** Association between the mRNA levels of LRP5 and the demographic and clinicopathological parameters of CRC patients in the Cancer Genome Atlas (TCGA)

Parameters	Groups	LRP5 Expression		χ^2^	*p*‐value
		Low	High		
Age	≤65	84	99	1.684	0.194
	>65	101	91		
Gender	Male	106	100	0.824	0.364
	Female	79	90		
TNM	T1/T2	34	34	0.015	0.903
	T3/T4	151	156		
	N0	110	98	2.357	0.125
	N1/N2/N3	75	92		
	M0	167	157	4.655	0.031
	M1	18	33		
Clinical stage	I/II	110	93	4.172	0.041
	III/IV	75	97		
History of colon polyps	No	108	120	2.231	0.135
	Yes	36	26		
BMI	<24	37	32	0.289	0.591
	≥24	64	65		
Living status	Living	144	145	0.311	0.578
	Dead	38	44		

**TABLE 3:** Univariate and multivariate analysis of OS and RFS of CRC patients

Parameters	Univariate analysis				Multivariate analysis	
	Groups	Mean survival time (Days)	Log rank χ^2^	*p*‐value	OR (95% CI)	*p*‐value
OS						
LRP5 Expression	Low	2646	0.139	0.710		
	High	2754				
TNM	T1/T2	2997	3.239	0.072	1.000	0.618
	T3/T4	2666			1.218 (0.561–2.645)	
	N0	2988	17.751	<0.001	1.000	0.882
	N1/N2/N3	2341			1.116 (0.263–4.739)	
	M0	2921	21.620	<0.001	1.000	0.003
	M1	1307			2.403 (1.359–4.248)	
Clinical Stage	I/II	2979	19.250	<0.001	1.000	0.348
	III/IV	2373			2.075 (0.452–9.514)	
Gender	Male	2340	0.692	0.405		
	Female	3023				
Age	≤65	3380	11.973	0.001	1.000	<0.001
	>65	2283			2.970 (1.827–4.829)	
History of colon polyps	No	2999	0.365	0.546		
	Yes	2994				
BMI	<24	2529	0.050	0.823		
	≥24	2857				
RFS						
LRP5 Expression	Low	2750	0.230	0.631		
	High	2711				
TNM	T1/T2	3195	7.441	0.006	1.000	0.080
	T3/T4	2628			2.314 (0.904–5.924)	
	N0	3052	18.924	<0.001	1.000	0.132
	N1/N2/N3	2197			3.304 (0.699–15.629)	
	M0	2953	21.676	<0.001	1.000	0.008
	M1	1179			2.226 (1.233–4.021)	
Clinical Stage	I/II	3049	18.258	<0.001	1.798 (0.363–8.908)	0.472
	III/IV	2205			1.000	
Gender	Male	2427	3.699	0.050	1.534 (0.970–2.426)	0.050
	Female	3016			1.000	
Age	≤65	2545	0.678	0.410		
	>65	2830				
History of colon polyps	No	2870	0.509	0.475		
	Yes	2537				
BMI	<24	2250	0.514	0.473		
	≥24	2648				

### 3.3 Activation of LRP5 promotes the tumorigenicity of CRC cells

**FIGURE 3:**
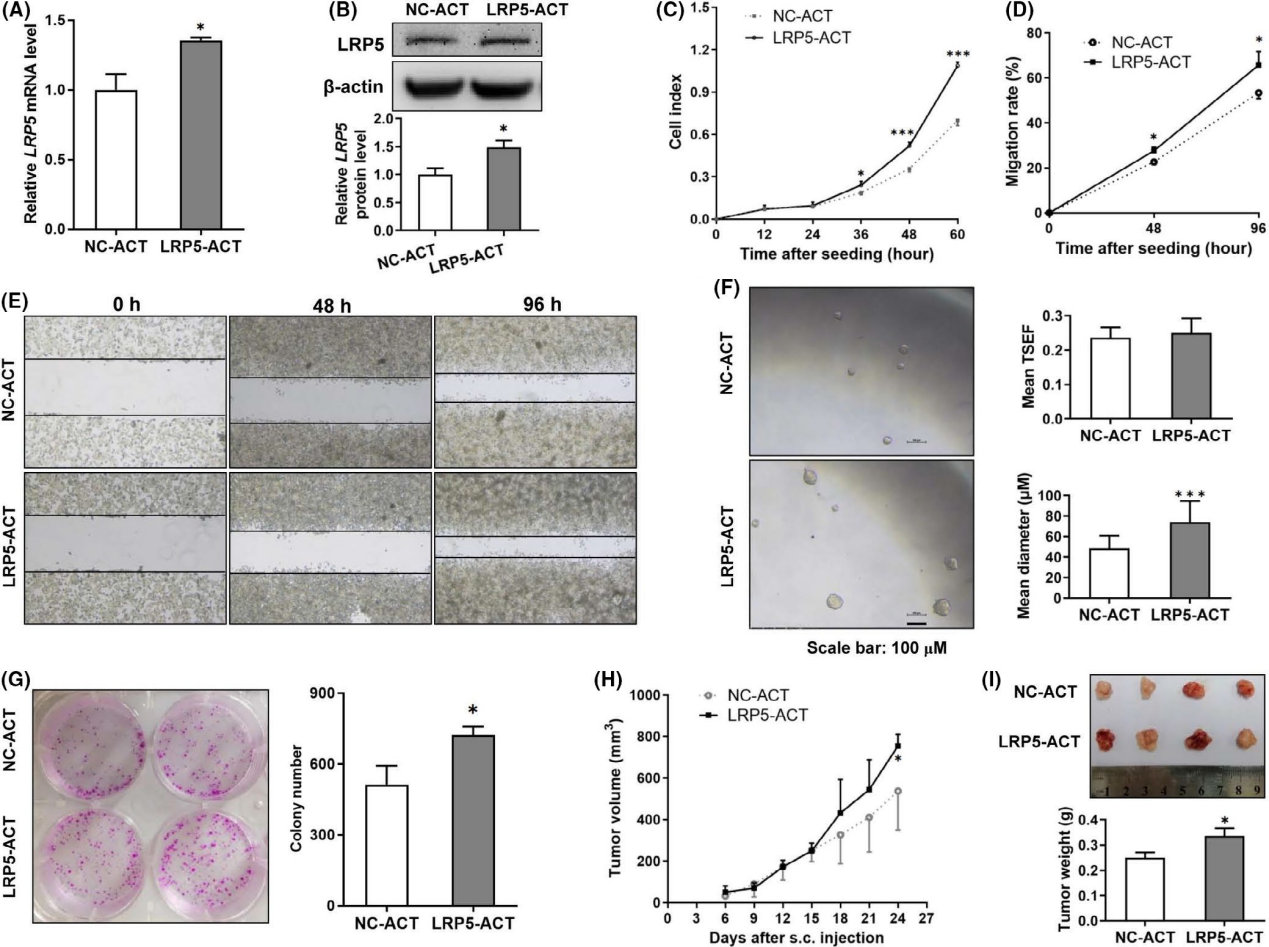
Activation of LRP5 accelerates the proliferation, migration and tumour growth of CRC cells. (A) Representative qRT‐PCR analysis of LRP5 mRNA level in NC‐ACT or LRP5‐ACT HCT‐116 cells. (B) Representative immunoblot showing LRP5 protein levels extracts from NC‐ACT or LRP5‐ACT HCT‐116 cells, and the ratio of LRP5 protein levels to β‐actin protein levels was quantified after densitometry. (C) The cell proliferation ability of LRP5‐ACT HCT‐116 cells and control ones was analysed by the real‐time cell analysis (RTCA) system, and the proliferation rates of cells at the different time points were analysed. (D‐E) Representative photographs (E) of NC‐ACT or LRP5‐ACT HCT‐116 cells taken at 0, 48, 96 h post‐wound (× 40), and their wound closure rates (D) were calculated. (F) Self‐renewal activity of NC‐ACT or LRP5‐ACT HCT‐116 cells was assessed by sphere‐forming assays. The mean TSFE and diameter of sphere was determined 12 days after the seeding. (G) Representative images of cell clones of NC‐ACT or LRP5‐ACT HCT‐116 cells, and the colony number was quantified by IPP 6.0 software. (H‐I) The real‐time tumour size (H), representative dissected tumours from nude mice and tumour weight (I) after sacrifice were shown. *p < 0.05, ***p < 0.001 vs. NC‐ACT group

To investigate whether activation of LRP5 has a potential carcinogenic effect on CRC cells, we used LRP5‐CRISPR activation plasmid to construct cell lines with stable activation of endogenous transcription of *LRP5* gene in HCT‐116 cells with puromycin selection. As shown in Figure 3A, B, both the transcriptional and translational levels of LRP5 were elevated in LRP5‐ACT group. To determine the effect of LRP5 activation on proliferative ability of CRC cells, the cellular proliferative indices were real‐time monitored by using the RTCA system. Activation of LRP5 obviously accelerated the proliferation of HCT‐116 cells (Figure 3C). We further performed the wound‐healing assay to test the effect of LRP5 activation on cellular migration. As shown in Figure 3D, E, activation of LRP5 led to a significant increase in migratory capacity. To explore the effect of LRP5 activation on self‐renewal capacity of CRC cells, a key feature of CSCs, we cultured the cells in serum‐free medium to generate typical tumourspheres. As shown in Figure 3F, LRP5‐ACT HCT‐116 cells formed bigger tumourspheres than control ones (Figure 3F). Subsequently, the tumoursphere‐formation capacity was determined by using colony formation assay. The results showed that LRP5‐ACT HCT‐116 cells formed approximately 1.5 times more tumourspheres than NC‐ACT ones (Figure 3G). To investigate whether activation of LRP5 could enhance the tumorigenic ability of CRC cells *in vivo*, nude mice were subcutaneously injected with LRP5‐ACT or NC‐ACT HCT‐116 cells respectively. The growth curve showed that LRP5‐ACT HCT‐116 cells could grow faster and form larger tumours than that of the NC‐ACT ones, suggesting activation of LRP5 obviously accelerated the tumour growth *in vivo* (Figure 3H, I). Collectively, these results show that activation of LRP5 exerts a tumour‐promoting role in the development of CRC, both *in vivo* and *in vitro*.

### 3.4 Activation of LRP5 promotes the stemness of CRC through activating the canonical Wnt/β‐catenin pathway and IL‐6/STA3 pathway

**FIGURE 4:**
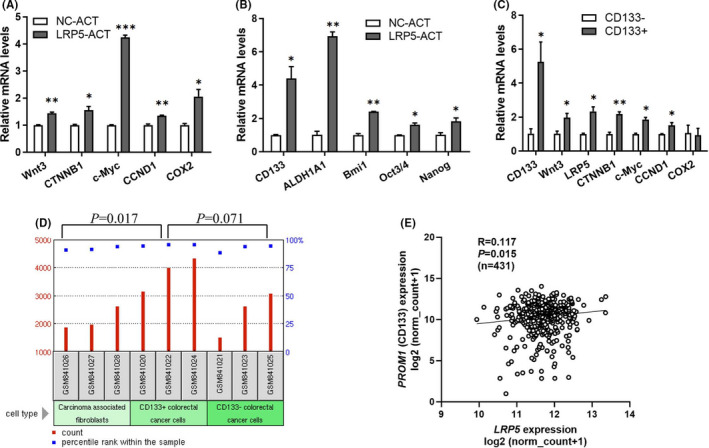
Activation of LRP5 upregulates the expression of crucial genes involved in canonical Wnt/β‐catenin signalling pathway and CSCs. (A‐B) The mRNA levels of crucial genes involved in canonical Wnt/β‐catenin signalling (A) and CSCs (B) in NC‐ACT or LRP5‐ACT HCT‐116 cells were determined by qRT‐PCR analysis. *p < 0.05, **p < 0.01, ***p < 0.001 vs. NC‐ACT group. (C) The mRNA levels of crucial genes involved in canonical Wnt/β‐catenin signalling in CD133^+^ HCT‐116 cells and CD133^−^ ones were determined by qRT‐PCR analysis. *p < 0.05, **p < 0.01 vs. CD133^−^ group. (D) A microarray data set (GSE34053) from the online GEO database was used to evaluate the LRP5 levels in CD133^+^ CRC cells, CD133^−^ cells and carcinoma‐associated fibroblasts isolated from a specimen of colon cancer patient in stage II. A *p*‐value lower than 0.05 was considered as statistically significant. (E) Significant positive correlation between LRP5 and PROM1 (encoding CD133) mRNA levels was observed in CRC tissues. Statistical significance was confirmed by correlation analysis using GraphPad 8.0 software

To reveal the underlying mechanism by which the activation of LRP5 promotes the progression of CRC, we analysed the change in some crucial genes in the canonical Wnt/β‐catenin pathway. As expected, the mRNA levels of Wnt3, CTNNB1, c‐Myc, CCND1 and COX2 were all upregulated (Figure 4A). It has been reported that CSCs endow CRC with some malignant properties and activating canonical Wnt/β‐catenin pathway could enhance the cell stemness,^32^ and we next explored whether activation of LRP5 could affect the stem‐like properties of CRC cells by activating the canonical Wnt/β‐catenin pathway. It was interesting to find that activation of LRP5 obviously upregulated the expression of several stemness‐related markers, including CD133, ALDH1A1, Bmi1, OCT3/4 and Nanog (Figure 4B). This might be the main reasons for the changes in cellular activities, including the enhanced tumoursphere‐formation and tumour growth capacities. Several studies have proved that canonical Wnt/β‐catenin pathway was overactive in colorectal CSCs. To confirm this conclusion, we specifically separated CD133‐positive (CD133^+^) cells and CD133‐negative (CD133^−^) ones from HCT‐116 cells by FACS sorting to analyse the changes of some crucial genes in canonical Wnt/β‐catenin pathway by qRT‐PCR. The results showed that the mRNA levels of Wnt3, LRP5, CTNNB1, c‐Myc and CCND1 were all upregulated in CD133^+^ HCT‐116 cells compared with those in CD133^−^ ones (Figure 4C). By analysing the GSE34053 data set,^33^ we further confirmed that LRP5 mRNA level was upregulated in CD133^+^ CRC cells, compared with that in CD133^−^ ones (*p *= 0.071) and carcinoma‐associated fibroblasts (*p *= 0.017) isolated from the same stage II colon cancer patient specimen (Figure 4D). Interestingly, we found an obviously positive correlation between the mRNA levels of LRP5 and PROM1 (CD133) (*p *= 0.015, R = 0.117) through analysing the data on CRC and normal tissues from the same TCGA database (Figure 4E), further confirmed the positive regulation of LRP5 activation on stemness of CRC.

**FIGURE 5:**
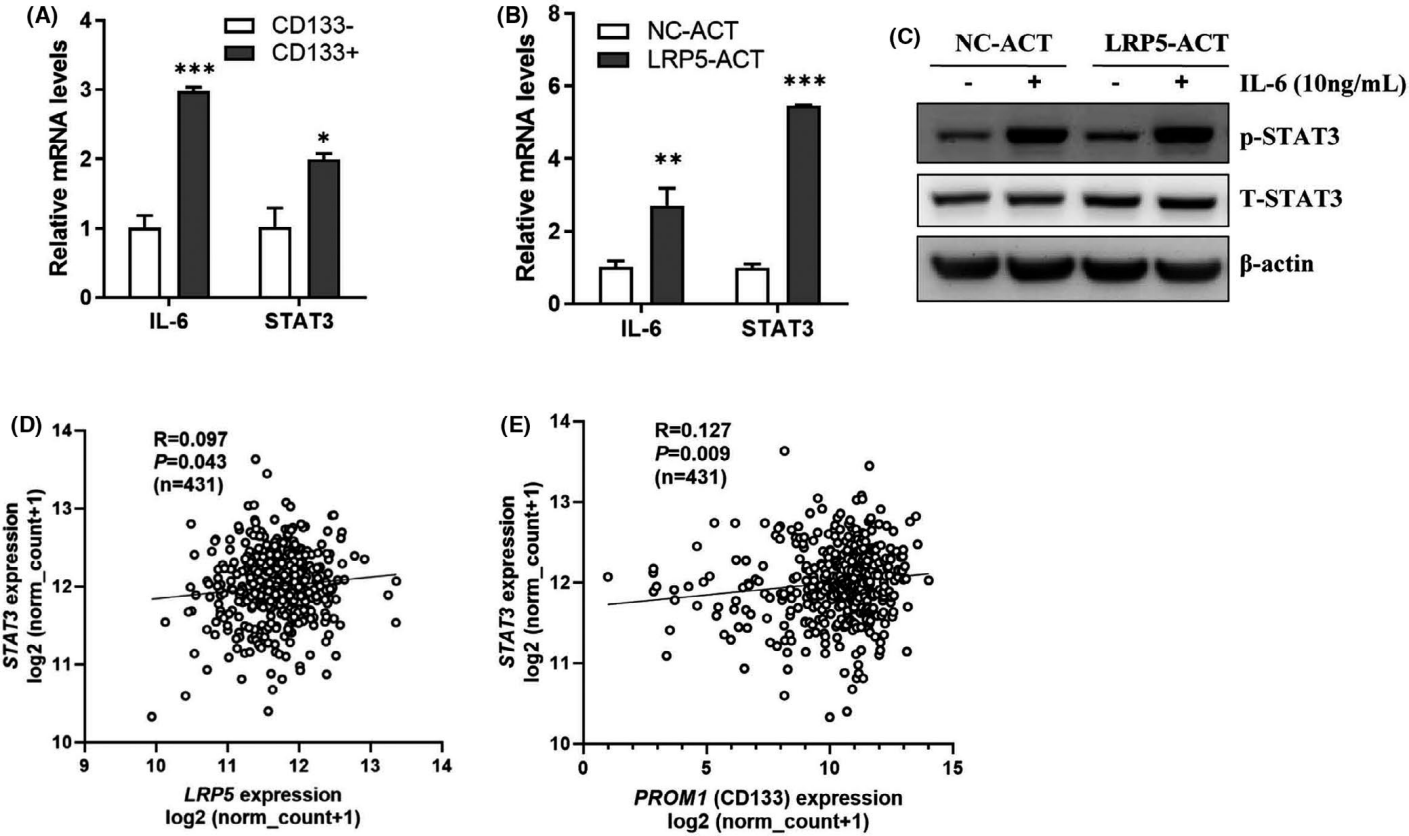
IL‐6/STA3 pathway is activated in LRP5‐ACT HCT‐116 cells and CD133^+^ HCT‐116 cells. (A) The mRNA levels of IL‐6 and STAT3 in CD133^+^ HCT‐116 cells and CD133^−^ ones were determined by qRT‐PCR analysis. *p < 0.05, ***p < 0.001 vs. CD133^−^ group. (B) The mRNA levels of IL‐6 and STAT3 in NC‐ACT or LRP5‐ACT HCT‐116 cells were determined by qRT‐PCR analysis. **p < 0.01, ***p < 0.001 vs. NC‐ACT group. (C) Total STAT3 (t‐STAT3) and phosphorylated STAT3 (p‐STAT3) protein levels in lysates extracted from NC‐ACT or LRP5‐ACT HCT‐116 cells treated with PBS or IL‐6 (10 ng/ml) for 1 h prior to harvest were determined by Western blot, β‐actin as a loading control. (D‐E) Significant positive correlation among LRP5, PROM1 (CD133) and STAT3 mRNA levels was observed in CRC tissues

The IL‐6/STAT3 inflammatory signalling axis was proved to promote stem‐like properties of CRC, and we further questioned whether the upregulation of stemness genes induced by the activation of LRP5 could be partially attributed to the activation of IL‐6/STAT3 pathway. As shown in Figure 5A, B, we were surprised to find that the mRNA levels of IL‐6 and STAT3 were both upregulated in LRP5 activation HCT‐116 cells and CD133^+^ ones, and LRP5 activation enhanced the phosphorylation of STAT3 (p‐STAT3) in HCT‐116 cells (Figure 5C). In addition, positive correlations were found between the mRNA levels of LRP5 and STAT3 (*p *= 0.043, R = 0.097), and between the mRNA levels of STAT3 and PROM1 (CD133) (*p *= 0.009, R = 0.127) (Figure 5D, E). Taken together, these results show that activation of LRP5 promotes the stemness of CRC, especially the CD133‐related stemness by activating canonical Wnt/β‐catenin and IL‐6/STA3 signalling pathways.

### 3.5 Activation of LRP5 induces the drug resistance of CRC cells to chemotherapeutic agents

**FIGURE 6:**
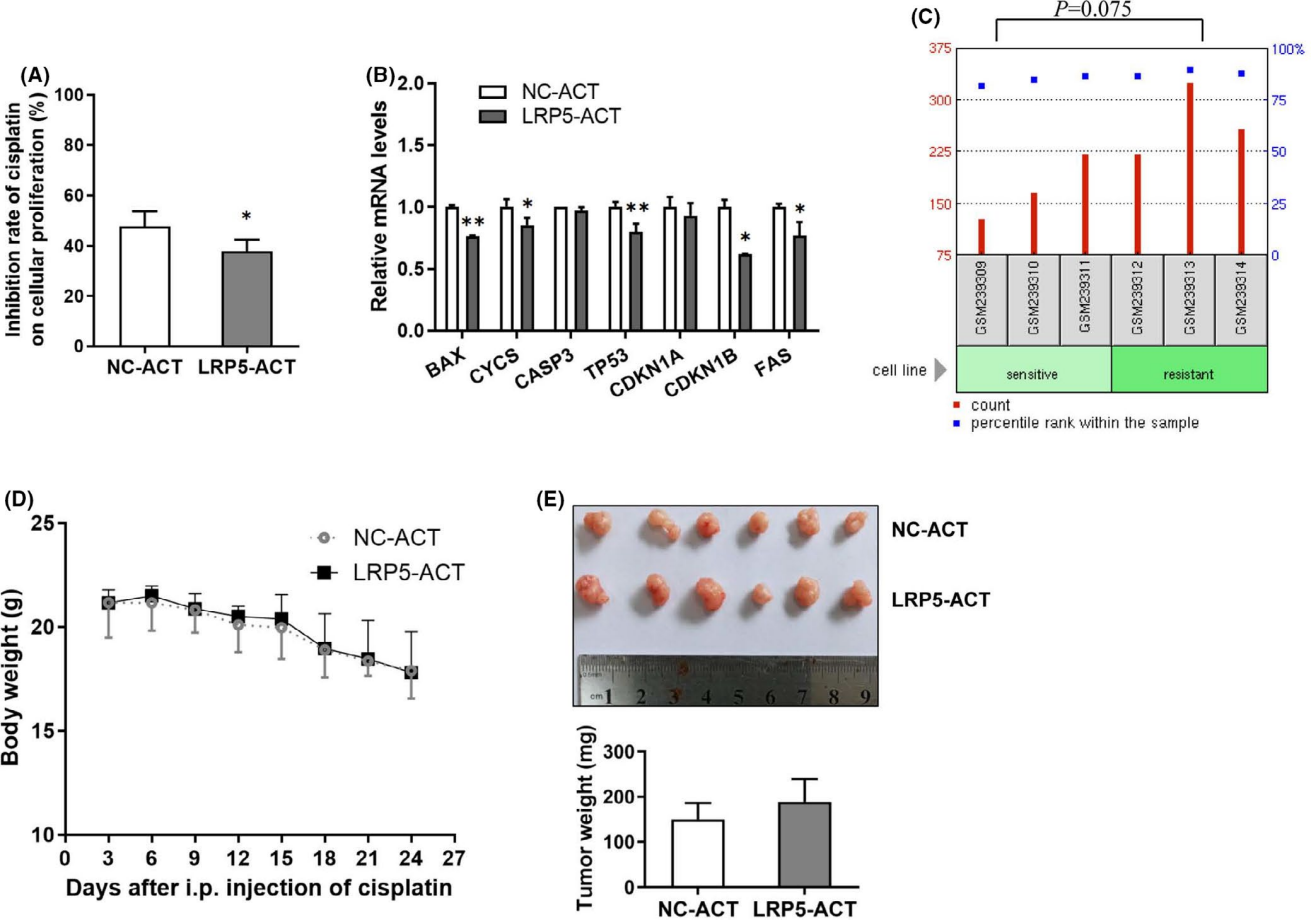
Activation of LRP5 reduces the sensitivity of CRC cells to chemotherapeutic agents. (A) The relative inhibition rate of cisplatin on cellular proliferation was determined in NC‐ACT or LRP5‐ACT HCT‐116 cells. Cells were treated with PBS or 12 μg/ml cisplatin for 24 h and cellular proliferation indices were monitored by RTCA, the inhibition rate was calculated according to the proliferation index of cells at the 24th hour. (B) The mRNA levels of pro‐apoptotic genes in NC‐ACT or LRP5‐ACT HCT‐116 cells treated with 12 μg/ml cisplatin for 24 h were determined by qRT‐PCR analysis. *p < 0.05, **p < 0.01 vs. NC‐ACT group. (C) The relative expression levels of LRP5 in the methotrexate‐resistant HT‐29 CRC cells and methotrexate‐sensitive ones in the GSE9412 data set. (D) The real‐time body weight of nude mice received i.p. treatment of 1 mg/kg cisplatin every three days for 24 days. (E) Representative dissected tumours from nude mice received cisplatin treatment and tumour weight after sacrifice were shown

The chemotherapeutic resistance is the key characteristics of CSCs and contributes to the postoperative recurrence and metastasis. Platinum‐based drugs are first‐line chemotherapeutic agents to treat CRC, and cisplatin was reported to inhibit the growth of xenograft tumours formed by HCT‐116 cells.^34^ To explore whether the activation of LRP5 could affect the resistance of CRC cells to chemotherapeutic agents, we treated LRP5‐ACT or NC‐ACT HCT‐116 cells with cisplatin and evaluated the cellular viability by RTCA system and calculated the inhibitory rate of cisplatin on cells. The results showed that CRC cells with the activation of LRP5 were more resistant to cisplatin than the control ones (Figure 6A). Subsequently, we explored the effect of LRP5 activation on the expression of apoptosis‐related genes after the treatment of cisplatin in CRC cells. As expected, the mRNA levels of some pro‐apoptotic genes, including *BAX*, *CYCS*, *TP53*, CDKN1B and *FAS*, were downregulated in LRP5‐ACT HCT‐116 cells compared with control cells (Figure 6B). Furthermore, we explored the expression level of LRP5 in drug‐resistant CRC cells by analysing a treatment‐related GEO microarray data set. In the GSE9412 data set,^35^ we found that LRP5 mRNA level was upregulated in methotrexate‐resistant HT‐29 CRC cells compared with sensitive ones, even though the statistical difference was not significant (*p *= 0.075) (Figure 6C). To evaluate the effect of LRP5 activation on the drug resistance of CRC cells to chemotherapeutic agents in vivo, nude mice implanted with LRP5‐ACT HCT‐116 cells or control ones received cisplatin‐based chemotherapy. As shown in Figure 6D, long‐term administration of cisplatin caused the body weight loss of the nude mice, indicated the potential cytotoxicity of cisplatin *in vivo*. Although the tumour weight in LRP5 activation group was a little heavier than that in the control group after the treatment of cisplatin (Figure 6E), this difference was not statistically significant and needs further investigation. Together, these data suggested that activation of LRP5 induces the resistance of CRC cells to chemotherapeutic agents.

### 3.6 Silencing of LRP5 suppresses the tumorigenicity of CRC cells

**FIGURE 7:**
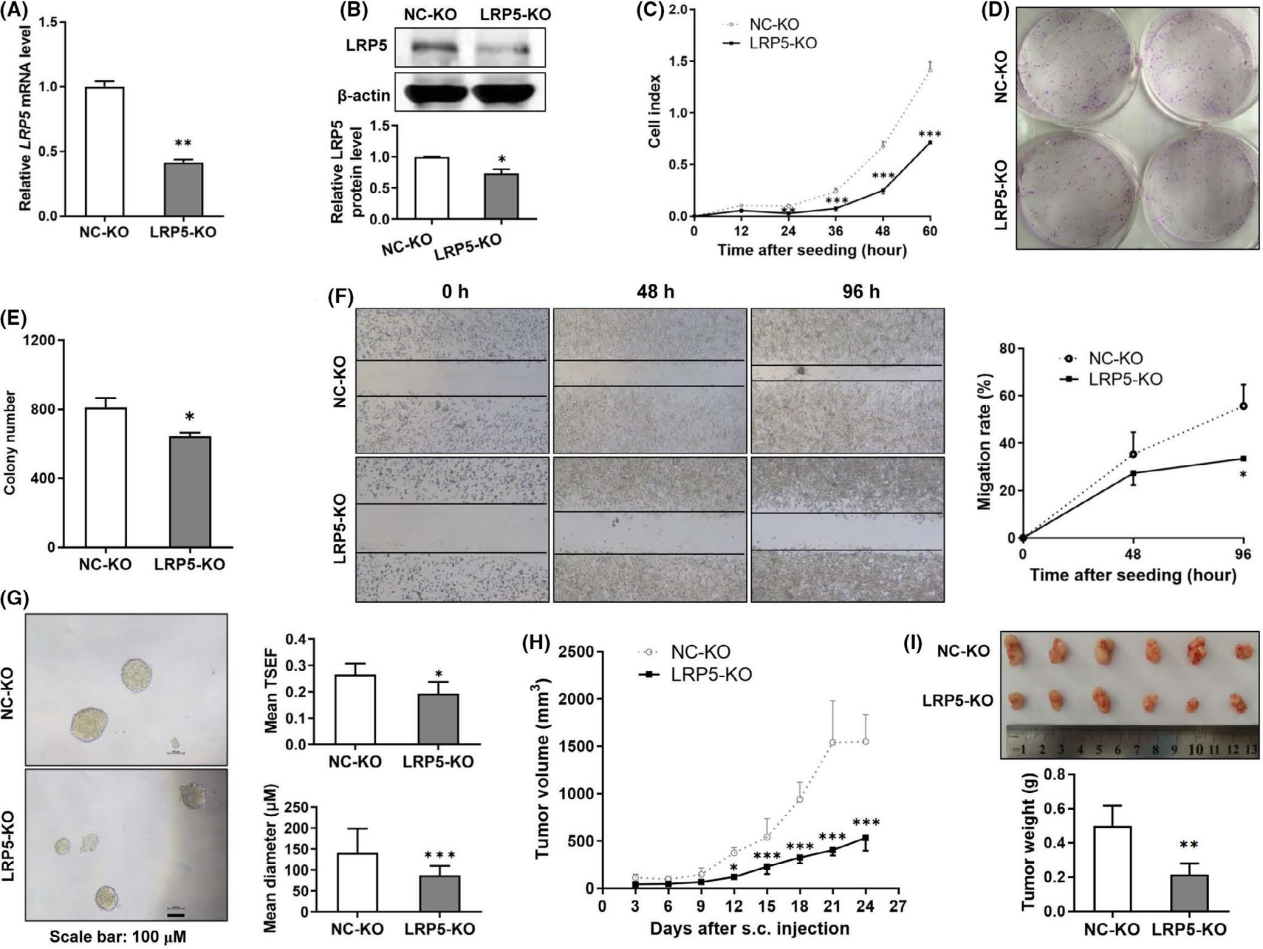
Knockdown of LRP5 inhibits the proliferation, migration and tumour growth of CRC cells. (A) The LRP5 mRNA levels in NC‐KO or LRP5‐KO HCT‐116 cells were determined by qRT‐PCR analysis. (B) Representative immunoblot showing LRP5 protein levels extracts from NC‐KO or LRP5‐KO HCT‐116 cells, and the ratio of LRP5 protein levels to β‐actin protein levels was quantified after densitometry. (C) The cell proliferation ability of LRP5‐KO HCT‐116 cells and control ones was analysed by the Real‐time cell analysis (RTCA) system, and the proliferation rates of cells at the different time point were analysed. (D‐E) Representative images (D) of cell clones of NC‐KO or LRP5‐KO HCT‐116 cells, and the colony number (E) was quantified by IPP 6.0 software. (F) Representative photographs of NC‐KO or LRP5‐KO HCT‐116 cells taken at 0, 48, 96 post‐wound (× 40), and their wound closure rates were calculated. (G) Self‐renewal activity of NC‐KO or LRP5‐KO HCT‐116 cells was assessed by sphere‐forming assays. The mean TSFE and diameter of sphere was determined 16 days after the seeding. (H‐I) The real‐time tumour size (H), representative dissected tumours from nude mice and tumour weight (I) after sacrifice were shown. *p < 0.05, **p < 0.01, ***p < 0.001 vs. NC‐KO group

Since LRP5 has a carcinogenic role in CRC development, we explored whether silencing the endogenous *LRP5* gene may exert an opposite effect. We constructed a stable LRP5‐KO HCT‐116 cell line. The results from qRT‐PCR and immunoblot analysis showed that the LRP5 mRNA and protein levels in LRP5‐KO cells were both decreased obviously (Figure 7A, B). An obvious inhibition of proliferative ability was found in LRP5‐KO cells compared with control ones (Figure 7C), indicated that silencing of LRP5 could suppress the cell proliferation of CRC cells. Moreover, silencing of LRP5 inhibited the colony formation and migratory capacities of CRC cells (Figure 7D–F), less and smaller tumourspheres were formed by LRP5‐KO HCT‐116 cells than control ones (Figure 7G). To further determine whether inhibition of LRP5 could regulate the tumorigenesis *in vivo*, LRP5‐KO HCT‐116 cells or control ones were transplanted into nude mice and the result showed that silencing of LRP5 indeed suppressed CRC cell tumorigenicity (Figure 7H, I). Together, these data suggest that silencing of LRP5 inhibits the tumorigenicity of CRC cells, both *in vitro* and *in vivo*.

### 3.7 Silencing of LRP5 suppresses the stemness of CRC by inhibiting the Canonical Wnt/β‐catenin pathway and IL‐6/STA3 pathway

**FIGURE 8:**
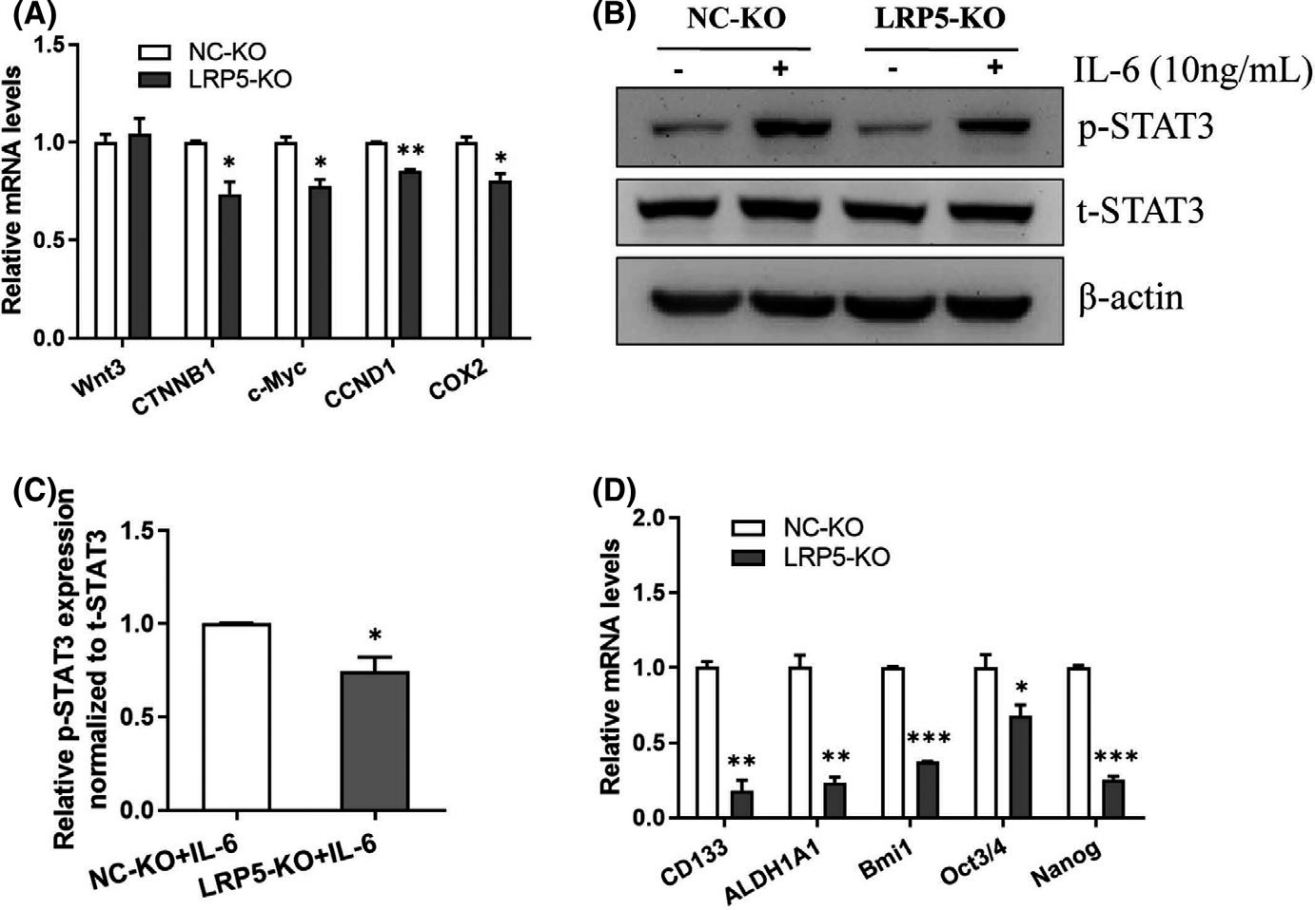
Knockdown of LRP5 inhibits the expression of crucial genes involved in canonical Wnt/β‐catenin pathway, CSCs and IL‐6/STA3 pathway. (A) The mRNA levels of crucial genes involved in canonical Wnt/β‐catenin pathway in NC‐KO or LRP5‐KO HCT‐116 cells were determined by qRT‐PCR analysis. *p < 0.05, **p < 0.01 vs. NC‐KO group. (B) Total STAT3 (t‐STAT3) and phosphorylated STAT3 (p‐STAT3) protein levels in lysates extracts from NC‐KO or LRP5‐KO HCT‐116 cells treated with PBS or IL‐6 (10 ng/ml) for 1 h prior to harvest were determined by Western blot, β‐actin as a loading control. (C) Densitometry was used to quantify relative p‐STAT3 protein levels normalized to t‐STAT3 protein levels. *p < 0.05 vs. NC‐KO+IL‐6 group. (D) The mRNA levels of CSCs‐related genes in NC‐KO or LRP5‐KO HCT‐116 cells were determined by qRT‐PCR analysis. *p < 0.05, **p < 0.01, ***p < 0.001 vs. NC‐KO group

We further explored the effect of LRP5 knockdown on the canonical Wnt/β‐catenin pathway. As shown in Figure 8A, knockdown of LRP5 inhibited the mRNA levels of some crucial genes in this cascade, including *CTNNB1*, *c*‐*Myc*, *CCND1* and *COX2*. In contrast, the expression of its upstream molecule, such as Wnt3, was not affected. We then examined the effect of LRP5 knockdown on the signal transduction of IL‐6/STAT3 pathway in CRC cells. Contrary to the LRP5 activation, silencing of LRP5 inhibited the p‐STAT3 expression levels induced by IL‐6 (Figure 8B, C). As shown in Figure 8D, the expression of stemness‐related markers, including CD133, ALDH1A1, Bmi1, Oct3/4 and Nanog, was inhibited dramatically, which might be the main reason for the downregulation of cell proliferation and tumour growth capacities. Collectively, these results suggest that silencing of LRP5 suppresses the stemness of CRC by inhibiting the canonical Wnt/β‐catenin and IL‐6/STA3 pathways simultaneously, and targeting the overexpressed LRP5 could be a valuable strategy for CRC treatment.

### 3.8 Silencing of LRP5 sensitizes CRC cells to cisplatin and induces cell apoptosis

**FIGURE 9:**
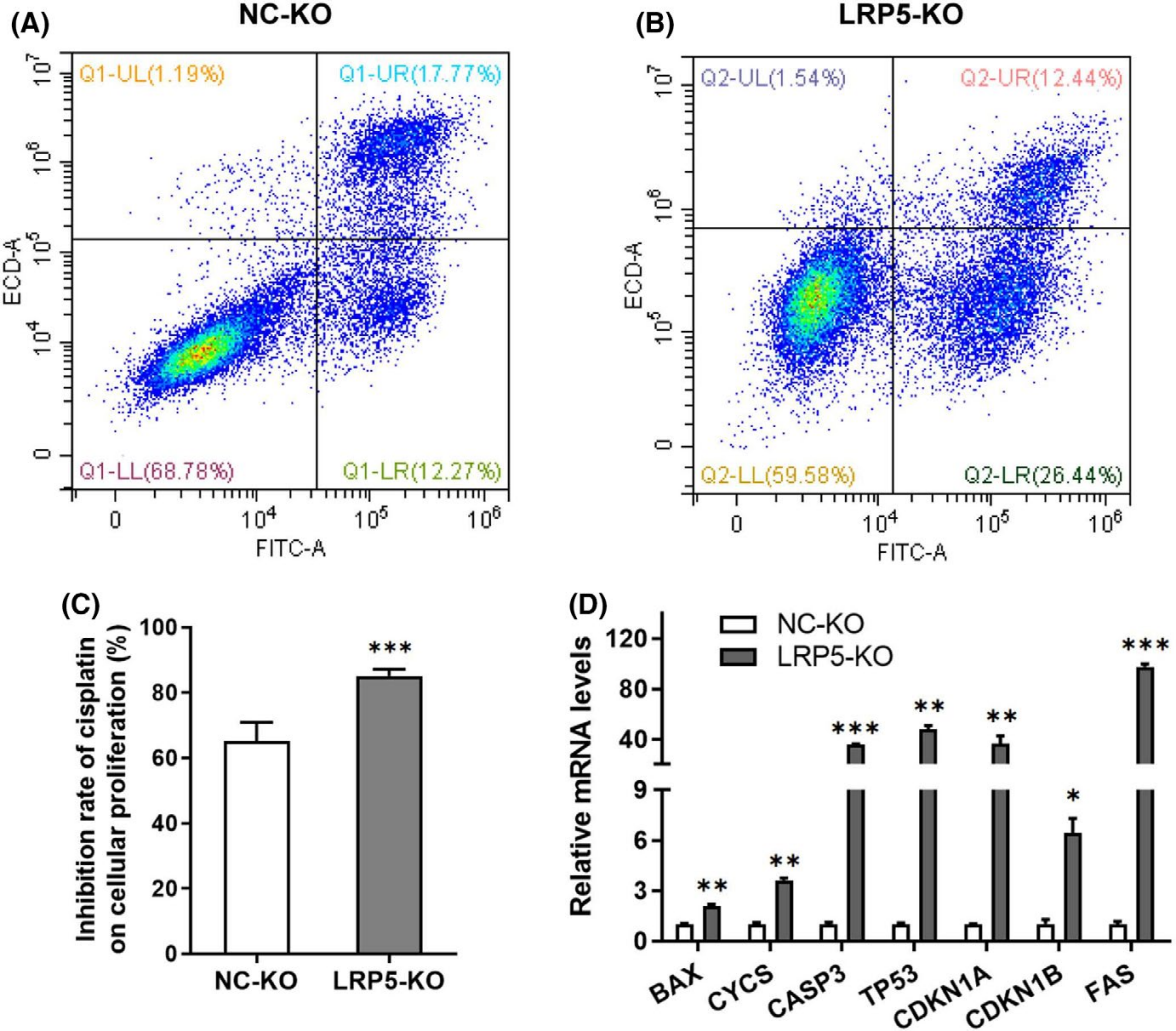
Knockdown of LRP5 improves the sensitivity of CRC cells to chemotherapeutic agents. (A) The apoptotic rate of NC‐KO or LRP5‐KO HCT‐116 cells treated with 8 μg/ml cisplatin for 36 h was analysed by flow cytometry. (B) The relative inhibition rate of cisplatin on cellular proliferation was determined according to the proliferation index of NC‐KO or LRP5‐KO HCT‐116 cells treated with 8 μg/ml at the 36th hour. (C) The mRNA levels of pro‐apoptotic genes in NC‐KO or LRP5‐KO HCT‐116 cells treated with 8 μg/ml cisplatin for 36 h were determined by qRT‐PCR analysis. *p < 0.05, **p < 0.01, ***p < 0.001 vs. NC‐KO group

We proceeded to assess the effect of LRP5 knockdown on the chemotherapeutic sensitivity of CRC cells. The data from the flow cytometry analysis showed that a higher proportion of early‐stage apoptotic cells was detected in LRP5‐KO group (26.44%), compared with that in NC‐KO group (12.27%) (Figure 9A). To explore whether silencing of LRP5 could enhance the sensitivity of CRC cells to the treatment of cisplatin, proliferation indices of cells exposed to cisplatin were real‐time monitored via RTCA system. As expected, LRP5‐KO HCT‐116 cells were more sensitive to cisplatin treatment than the control ones (Figure 9B). Subsequently, we explored the effect of LRP5 knockdown on the expression of pro‐apoptotic genes through the induction of cisplatin in CRC cells. As expected, the mRNA levels of pro‐apoptotic genes, including *BAX*, *CYCS*, *CASP3*, *TP53*, *CDKN1A*, *CDKN1B* and *FAS*, were all significantly upregulated in LRP5‐KO HCT‐116 cell (Figure 9C). Taken together, these results illustrate that silencing of LRP5 promotes the sensitivity of CRC cells to chemotherapeutic agent and its induced cell apoptosis, and targeting LRP5 could be a valuable strategy for CRC treatment.

## 4 DISCUSSION

At present, the role of canonical Wnt/β‐catenin signalling pathway in the initiation and progression of malignancies has been determined and attracted much attention, whereas the components of this pathway are complicated and their functions in specific type of cancer remain ambiguous. LRP5 is a crucial component of the LRP5/6/FZDs co‐receptor group in canonical Wnt/β‐catenin signalling pathway. Upon binding to Wnt ligands, FZDs and LRP5/6 undergo the polymerization to elicit the Wnt/β‐catenin signalling.^36^ Recent studies proved that oligomerizations of FZDs and LRP5/6 also caused a significant activation of ligand‐independent LRP5/β‐catenin signalling,^37^ and suppression of membranous LRP5 recycling and subsequent increasing of LRP5 lysosomal degradation by overexpressing 15‐Lipoxygenase‐1 (15‐LOX‐1), an important LA‐metabolizing enzyme, exerts obvious inhibitory effect on CRC via silencing LRP5‐mediated canonical Wnt/β‐catenin pathway.^28^ A series of studies have documented the associations of LRP5 with several types of human malignancies, including osteosarcoma, leukaemia, prostate, parathyroid, breast and gastric cancers.^26^, ^38^, ^39^, ^40^, ^41^, ^42^ However, the expression pattern and role of LRP5 in different tumour microenvironment are not always consistent. For instance, LRP5 is overexpressed in high‐grade osteosarcoma, and inhibiting the canonical Wnt/β‐catenin pathway by using a soluble dominant‐negative LRP5 could suppress the invasive capacity and epithelial to mesenchymal transition (EMT) in osteosarcoma cells.^43^, ^44^ Analogously, silencing of LRP5 in prostate cancer cells leads to a robust decrease in invasion growth and skeletal metastasis *in vitro* and *in vivo* by decreasing the expression of pro‐invasive and pro‐metastatic genes.^26^ However, the role of LRP5 demonstrates some discrepancies in breast cancer. Ren et al. found that LRP5/6 could directly bind to FZDs to prevent the activation of non‐canonical pathway and tumour metastasis in mouse and human breast cancer.^45^ In contradiction to this finding, another study reported that LRP5 was overexpressed in triple‐negative breast cancers and LRP*5* depletion decreased tumorigenesis and induced the apoptosis of cancer cells.^42^ Therefore, the role of LRP5 in the progression of cancer is complicated and may depend on specific tumour microenvironment or the heterogeneity of cancers.

The expression of LRP5 in human CRC has never been determined as yet, and we therefore compared its expression level in CRC tissues and matched normal colorectal ones by qRT‐PCR analysis. The results showed that LRP5 mRNA levels were upregulated in CRC tissues and some CRC cell lines, such as Caco2 and HCT‐116. However, those in LoVo and SW480 cells were not elevated, probably because these two cell lines were established from metastatic tumours of CRC and had different genetic background. By analysing CRC‐related TCGA database and online HPA, we were more convinced LRP5 was upregulated in CRC tissues at both mRNA and protein levels. However, the increasing amplitude of LRP5 in qRT‐PCR results was significantly greater than that in Oncomine analysis. A reason for the discrepancy is that the former is derived from analysing the paired colorectal tissues, but the latter is achieved from non‐paired tissues, and we speculated the data from paired tissues was much more accurate. Additionally, we found the upregulation of LRP5 was positively related to the advanced pathological stages and metastasis, whereas it had no correlation with OS and RFS. By performing the univariate survival analysis and multivariate analysis, we found metastasis, age over 65 years and male were independent prognostic indicators of poor OS or RFS in CRC. In contrast, we did not find any difference between the LRP5 expression and the disease prognosis, although LRP5 might be a valuable biomarker for the diagnosis of CRC.

Several studies have illustrated LRP5 is essential for the activation of canonical Wnt/β‐catenin signalling pathway and gives rise to the malignant transformation of normal cells and excessive proliferation of cancer cells.^46^ Nonetheless, the role of LRP5 in the tumorigenesis of CRC is still unclear. In the present study, we confirmed that activation of LRP5 in CRC cells increased the cellular proliferation, migration and enhanced chemoresistance *in vitro*, and promoted the tumour growth *in vivo* via activating the canonical Wnt/β‐catenin pathway. As expected, opposite conclusions were obtained when *LRP5* gene was silenced, strongly verified the tumorigenic role of LRP5 in colorectal carcinogenesis. CSCs are more resistant to chemoradiation therapy and lead to the malignant progression, metastasis, recurrence and poor prognosis by regenerating and maintaining the heterogeneous phenotypes of tumours.^5^, ^47^ Abundant studies have revealed the intimate relation between the Wnt signalling and CSCs.^46^, ^48^ Activating Wnt signalling by MEK inhibitors or overexpression of the heat shock protein molecular chaperone, TRAP1, could induce the plasticity and clonogenicity of CSCs in CRC,^49^, ^50^ in turn, overexpression of stemness‐related genes such as Oct4 drives Wnt/β‐catenin activation.^51^ As a cholesterol‐binding protein, CD133 was identified as an important CSC‐specific maker and determined to be responsible for the activation of Wnt signalling by preventing the acetylation and subsequent degradation of β‐catenin.^52^ Meanwhile, it was suppressed when silencing the Wnt signalling pathway by Celecoxib in CRC cells.^53^ However, the mechanism by which the Wnt signalling and CD133 have the ability to regulate each other remains unknown. In this study, we confirmed the canonical Wnt/β‐catenin signalling pathway was overactivated in CD133^+^ CRC cells, and firstly documented that the expression of LRP5 was upregulated in CD133^+^ CRC cells. Interestingly, the analyses on CRC‐based GEO data set also showed it was obviously increased in CD133^+^ CRC cells as compared to that in carcinoma‐associated fibroblasts, a heterogeneous cell population arised from tumour‐infiltrating mesenchymal stem cells or resting fibroblasts and provide a tumour‐promoting microenvironment for tumour progression and metastasis.^54^ An obvious positive correlation was also found between the mRNA levels of LRP5 and PROM1 (CD133) in CRC tissues. These data suggested that the overactivation of canonical Wnt/β‐catenin signalling in colorectal CSCs could be attributed to the upregulation of LRP5. Activation of LRP5 also promoted the self‐renewal capacity of CSCs by upregulating the expression of stemness‐related genes, including *PROM1* (CD133). We speculate that the activation of canonical Wnt/β‐catenin signalling induced by LRP5 overexpression expands the CSCs population and strengthens their stemness‐related functions, which further upregulate LRP5 expression and eventually forms a vicious circle. Therefore, LRP5 is the intermediary factor that mediates the mutual activation between the canonical Wnt signalling and the stemness of CSCs in CRC. Angiogenesis also plays an important role in the progression of CRC, and CSCs derived from CRC can generate functional cancer blood vessels and enhance tumour neovascularization by expressing some angiogenic factors.^55^ The expression level of certain well‐known human CSC marker, such as CD133, is also positively associated with the angiogenesis of CRC.^56^ Therefore, the activation of LRP5 might induce the angiogenesis of CRC by facilitating the self‐renewal of CSCs and needs further investigation. Our data also suggested that LRP5 was a promising target for treating CRC through eliminating CSCs. Unfortunately, due to the lower expression level of CD133 protein on the surface of other CRC cells, it is difficult to sort CD133^+^ cells except from HCT‐116 cells and results based on a single cell line are inevitably limited. Similarly, a recent study reported single‐domain antibody fragments binding to LRP6 strongly inhibited the growth of Wnt‐hypersensitive intestinal stem cells through stem cell exhaustion and promoting terminal differentiation provides a promising strategy for treating Wnt‐hypersensitive tumours.^57^ Strikingly, Lettini and colleagues just found the expression of LRP5 was positively regulated by TRAP1 at transcriptional level by promoter methylation mechanisms, and TRAP1 was upregulated in 60–70% human CRC. Therefore, TRAP1‐induced overexpression of LRP5 might be responsible for the excessive activation of canonical Wnt/β‐Catenin signalling and stemness maintenance in CSCs of human CRC.^58^

It was reported that inducing IL‐6/STAT3 inflammatory pathway via silencing claudin‐3 expression promoted CRC malignancy by overactivating Wnt/β‐catenin pathway,^59^ and IL‐6 could promote the EMT of CRC cells through activating the canonical Wnt/β‐catenin signalling pathway in STAT3/ERK‐dependent.^60^ However, it is not clear whether the IL‐6/STAT3 pathway is regulated by the canonical Wnt/β‐catenin pathway in the malignant progression of CRC and other cancers. As shown above, activation of LRP5 increased the mRNA levels of IL‐6 and STAT3, and the phosphorylation of STAT3 protein. On the contrary, the phosphorylation of STAT3 induced by IL‐6 was suppressed in LRP5 knockdown CRC cells. Recently, a study documented that activation of IL‐6/STAT3 signalling pathway served as an independent tumorigenic factor for the CRC via promoting stem‐like properties.^61^ In lung cancer, activation of Akt1/IL‐6/STAT3 pathway also contributes to maintaining the stemness of tumour initiating cells.^62^ Therefore, we speculated that the enhancement of stemness phenotype induced by LRP5 activation might be partly ascribed to the overactivation of IL‐6/STAT3 signalling. In order to explore this hypothesis, we analysed the relationship among the expression of *LRP5*, *STAT3* and *PROM1* (CD133) genes in TCGA database. As expected, we found the mRNA levels of LRP5 and PROM1 (CD133) were both positively associated with the mRNA levels of STAT3. We subsequently treated the LRP5‐KO CRC cells with exogenous IL‐6 protein. However, the downregulation of the expression of stemness‐related genes, induced by silencing of LRP5, was not reversed after the IL‐6 treatment (data not shown). Taken together, these findings suggest that silencing of LRP5 could inhibit CSC‐like phenotype by blocking the canonical Wnt/β‐catenin and IL‐6/STAT3 pathways, and targeting the overexpressed LRP5 might be a promising therapeutic approach for CRC.

## 5 CONCLUSIONS

In summary, we demonstrated that the expression of LRP5 was upregulated in CRC tissues and cells, and activation of LRP5 exerted a carcinogenic effect on the progression of CRC. On the contrary, silencing of LRP5 suppressed the tumorigenicity and sensitized CRC to cisplatin treatment by targeting Wnt/β‐catenin mediated CSC properties and IL‐6/STAT3 pathway. Therefore, these findings broaden our knowledge of CRC development and provide a valuable therapeutic target for CRC research.

## CONFLICTS OF INTEREST

The authors have no conflicts of interest to disclose in relation to this article.

## AUTHOR CONTRIBUTIONS

**Xiaobo Nie:** Conceptualization (lead); Funding acquisition (equal); Project administration (lead); Supervision (equal); Writing – original draft (lead); Writing – review & editing (lead). **Huiyang Liu:** Methodology (equal); Software (equal). **Wenling Ye:** Methodology (equal). **Xiaoyun Wei:** Methodology (equal); Software (supporting). **Lili Fan:** Formal analysis (supporting); Methodology (supporting). **Han Ma:** Formal analysis (supporting); Methodology (supporting). **Lanqing Li:** Methodology (supporting); Visualization (supporting); Writing – review & editing (supporting). **Wanting Xue:** Investigation (equal); Methodology (equal). **Wenting Qi:** Investigation (equal); Methodology (equal). **Yan‐Dong Wang:** Methodology (equal). **Wei‐Dong Chen:** Conceptualization (equal); Funding acquisition (equal); Project administration (equal); Supervision (equal).

## ETHICS STATEMENT

This study was performed according to the recommendations of the Biomedical Research Ethics Committee of Henan University.

## Supporting information

Table S1
