# Supplementary material for: LRP5 promotes cancer stem cell traits and chemoresistance in colorectal cancer
Source: J Cell Mol Med. 2022 Jan 7;26(4):1095–112. doi: 10.1111/jcmm.17164 (PMC8831954; doi:10.1111/jcmm.17164)
Supplement: Supplementary file 1 — Table S1 [file JCMM-26-1095-s001.docx]

**Supplementary Table 1. The sequences of primers for human (h) genes used in qRT-PCR.**

| **Primer name** | **Sequences (5’-3’)** |
| --- | --- |
| h-actin-F | ACTGGGACGACATGGAGAAA |
| h-actin-R | CTGGATAGCAACGTACATGG |
| h-Wnt3-F | CCACAACACGAGGACGGAGA |
| h-Wnt3-F | CGCCCAGCCACACACTTC |
| h-LRP5-F | CCACAACACGAGGACGGAGA |
| h-LRP5-R | CGCCCAGCCACACACTTC |
| h-CTNNB1-F | GCGCCATTTTAAGCCTCTCG |
| h-CTNNB1-R | AAATACCCTCAGGGGAACAGG |
| h-CCND1-F | AGTTGCAAAGTCCTGGAGCC |
| h-CCND1-R | GTTTCCACTTCGCAGCACAG |
| h-c-Myc-F | GTCAAGAGGCGAACACACAAC |
| h-c-Myc-R | TTGGACGGACAGGATGTATGC |
| h-COX2-F | CTGGCGCTCAGCCATACAG |
| h-COX2-R | CGCACTTATACTGGTCAAATCCC |
| h-IL-6-F | CCTGAACCTTCCAAAGATGGC |
| h-IL-6-R | TTCACCAGGCAAGTCTCCTCA |
| h-STAT3-F | ATCACGCCTTCTACAGACTGC |
| h-STAT3-R | CATCCTGGAGATTCTCTACCACT |
| h-CD133-F | AGTCGGAAACTGGCAGATAGC |
| h-CD133-F | GGTAGTGTTGTACTGGGCCAAT |
| h-ALDH1A1-F | CTGCTGGCGACAATGGAGT |
| h-ALDH1A1-R | CGCAATGTTTTGATGCAGCCT |
| h-Bmi1-F | CCACCTGATGTGTGTGCTTTG |
| h-Bmi1-R | TTCAGTAGTGGTCTGGTCTTGT |
| h-Oct3/4-F | CTTGAATCCCGAATGGAAAGGG |
| h-Oct3/4-R | GTGTATATCCCAGGGTGATCCTC |
| h-Nanog-F | TTTGTGGGCCTGAAGAAAACT |
| h-Nanog-R | AGGGCTGTCCTGAATAAGCAG |
| h-CASP3-F | AGAGGGGATCGTTGTAGAAGTC |
| h-CASP3-F | ACAGTCCAGTTCTGTACCACG |
| h-TP53-F | CAGCACATGACGGAGGTTGT |
| h-TP53-R | TCATCCAAATACTCCACACGC |
| h-Bax-F | CCCGAGAGGTCTTTTTCCGAG |
| h-Bax-R | CCAGCCCATGATGGTTCTGAT |
| hsa-CYCS-F | TGGGCCAAATCTCCATGGTC |
| hsa-CYCS-R | AGGCAGTGGCCAATTATTACTCA |
| h-CDKN1A-F | TGTCCGTCAGAACCCATGC |
| h-CDKN1A-R | AAAGTCGAAGTTCCATCGCTC |
| h-CDKN1B-F | AACGTGCGAGTGTCTAACGG |
| h-CDKN1B-R | CCCTCTAGGGGTTTGTGATTCT |
| h-Fas-F | TCTGGTTCTTACGTCTGTTGC |
| h-Fas-R | CTGTGCAGTCCCTAGCTTTCC |
